# GHZ States as Tripartite PR Boxes: Classical Limit and Retrocausality

**DOI:** 10.3390/e20060478

**Published:** 2018-06-20

**Authors:** Daniel Rohrlich, Guy Hetzroni

**Affiliations:** 1 Department of Physics, Ben-Gurion University of the Negev, Beersheba 84105, Israel; 2 Program in the History and Philosophy of Science, The Hebrew University of Jerusalem, Jerusalem 91905, Israel

**Keywords:** axioms for quantum theory, PR box, nonlocal correlations, classical limit, retrocausality, 03.65.Ta, 03.65.Ca, 03.30.+p, 03.65.Ud

## Abstract

We review an argument that bipartite “PR-box” correlations, though designed to respect relativistic causality, in fact *violate* relativistic causality in the classical limit. As a test of this argument, we consider Greenberger–Horne–Zeilinger (GHZ) correlations as a tripartite version of PR-box correlations, and ask whether the argument extends to GHZ correlations. If it does—i.e., if it shows that GHZ correlations violate relativistic causality in the classical limit—then the argument must be incorrect (since GHZ correlations do respect relativistic causality in the classical limit.) However, we find that the argument does not extend to GHZ correlations. We also show that both PR-box correlations and GHZ correlations can be retrocausal, but the retrocausality of PR-box correlations leads to self-contradictory causal loops, while the retrocausality of GHZ correlations does not.

Quantum mechanics might make more sense to us if we could derive it from simple axioms with clear physical content, instead of opaque axioms about Hilbert space. Aharonov [1,2] and, independently, Shimony [3,4] conjectured that quantum mechanics might follow from the two axioms of nonlocality and relativistic causality (no superluminal signalling). For example, quantum correlations respect relativistic causality, but they are nonlocal: they violate the Bell-CHSH [5,6,7] inequality. Could quantum mechanics be *unique* in reconciling these axioms, just as the special theory of relativity is unique in reconciling the axioms of relativistic causality and the equivalence of inertial frames? So-called “PR-box” [8] correlations disprove this conjecture. Like quantum correlations, they respect relativistic causality; but unlike quantum correlations, they violate the Bell-CHSH inequality *maximally*. Nevertheless, Ref. [9] argues that the addition of one minimal axiom of clear physical content—namely, the existence of a classical limit—suffices for ruling out PR-box correlations.

The additional axiom is minimal in the following sense: Quantum mechanics has a classical limit in which there are no uncertainty relations; there are only jointly measurable macroscopic observables. This classical limit—our direct experience—is an inherent constraint, a boundary condition, on quantum mechanics and on any generalization of quantum mechanics. Thus PR-box correlations, too, must have a classical limit. Reference [9] argues that in this classical limit, PR-box correlations (and, by extension [10,11], all stronger-than-quantum bipartite correlations) allow observers “Alice” and “Bob” to exchange superluminal signals. (A similar statement appears in Ref. [12] with “macroscopic locality” taking the place of “classical limit”. Yet Ref. [12] assumes that Alice and Bob can detect fluctuations of order $\sqrt{N}$ in their measurements, an assumption we do not make.) The argument [9,10] relies on measurement sequences that are observable but exponentially improbable. It is therefore of interest to test the argument by applying it to a different problem. In particular, GHZ correlations [13] are a tripartite version of PR-box correlations in the sense of being all-or-nothing correlations (perfect correlations and anticorrelations). Could Alice, Bob and an additional observer, “Jim”, use GHZ correlations, in the classical limit, to exchange superluminal signals? Does the argument of Ref. [9] lead to this conclusion? If so, it is clearly an incorrect argument: quantum mechanics and its classical limit do *not* violate relativistic causality! The first section of this paper reviews the arguments of Ref. [9] and attempts to extend them to show how Alice, Bob and Jim could exchange superluminal signals in the classical limit; but this attempt fails. The second section compares PR-box and GHZ correlations to show how retrocausality is self-contradictory in the first case but not in the second.

## 1. GHZ and PR-Box Correlations in the Classical Limit

Let Alice and Bob make spacelike separated measurements on pairs of particles. For each pair (indexed by *i*), one member of the pair is in Alice’s laboratory, and she can choose to measure observables $a_{i}$ or $a_{i}^{\prime }$ (but not both) on it; the other member is in Bob’s laboratory, and Bob can choose to measure observables $b_{i}$ or $b_{i}^{\prime }$ (but not both) on it. All four observables $a_{i}$, $a_{i}^{\prime }$, $b_{i}$ and $b_{i}^{\prime }$ take values ±1 with equal probability. The definition of PR-box correlations,

(1) $$C\left(a_{i},b_{i}\right)=C\left(a_{i},b_{i}^{\prime }\right)=C\left(a_{i}^{\prime },b_{i}\right)=1=-C\left(a_{i}^{\prime },b_{i}^{\prime }\right),$$

implies that Alice can manipulate the correlations between the observables $b_{i}$, $b_{i}^{\prime }$ of Bob’s particle by choosing whether to measure $a_{i}$ or $a_{i}^{\prime }$: indeed, $b_{i}$ and $b_{i}^{\prime }$ are perfectly correlated if she measures $a_{i}$ (as both of them are perfectly correlated with her outcome), and perfectly anticorrelated if she measured $a_{i}^{\prime }$ (as $b_{i}$ is correlated with her outcome and $b_{i}^{\prime }$ is anticorrelated with it). Thus, even though Alice’s choice of measurement does not affect Bob’s distribution of either $b_{i}$ or $b_{i}^{\prime }$, it does affect correlations between these two observables. So can Alice exploit these correlations to signal to Bob? No, she cannot, since, by assumption, $b_{i}$ and $b_{i}^{\prime }$ are incompatible and Bob cannot measure both. But, notably, this assumption cannot apply in the classical limit.

Following Ref. [9], we define the classical limit of PR-box correlations as follows: Macroscopic (classical) quantities are averages over arbitrarily large ensembles of microscopic observables. To see how this definition applies, let us consider an ensemble of *N* pairs shared by Alice and Bob and obeying Equation (1). Apparently, the *N* pairs are just as useless for signalling as one pair, since, for each pair, Bob is allowed to measure only $b_{i}$ or $b_{i}^{\prime }$. But the classical limit as defined means that given a large enough ensemble, Bob can measure quantities which depend upon macroscopic averages such as $B=\sum_{i=1}^{N}b_{i}/N$ and $B^{\prime }=\sum_{i=1}^{N}b_{i}^{\prime }/N$, obtaining *some* information about both of them. There is no fundamental limit on how many times Alice and Bob can repeat their measurements, hence no matter how large they choose *N* (so as to minimize the variances in *B* and $B^{\prime }$), there is no limit to the strength of the (anti-)correlations that they may observe.

Now let us imagine two possible scenarios. In one scenario, Alice measures $a_{i}$ consistently on all her *N* particles. In the other scenario, she measures $a_{i}^{\prime }$ consistently on all her *N* particles. What does Bob obtain from his measurements? The average value of *B* is 〈B〉=0. Even typical deviations of *B* are small, i.e., of order $1/\sqrt{N}$, so they disappear in the classical limit. Apparently the scenarios lead to the exact same conclusion: Bob cannot read Alice’s 1-bit message, encoded in her choice of what to measure.

Yet it will sometimes happen (with probability $2^{-N}$) that *B* will take the value 1. If Alice and Bob repeat either scenario exponentially many times, they can produce arbitrarily many cases of B=1. True, there will be measurement errors in Bob’s results, but in the classical limit Bob must obtain at least some information about both *B* and $B^{\prime }$. Now if Alice consistently measures $a_{i}$, Bob can expect to obtain B=1 with probability close to $2^{-N}$. And he can also expect to obtain $B=1=B^{\prime }$ with the same probability, and not with probability $2^{-2N}$, because Alice’s choice has correlated 〈B〉 with $\langle B^{\prime }\rangle$. Conversely, if Alice consistently measures $a_{j}^{\prime }$, then Bob can expect to obtain B=1 with probability close to $2^{-N}$, and he can also expect to obtain $B=1=-B^{\prime }$ with the same probability, and not with probability $2^{-2N}$, because Alice’s choice has *anti*correlated 〈B〉 with $\langle B^{\prime }\rangle$. Another way for Bob to get Alice’s message is to observe the variance in his measurements of $B\pm B^{\prime }$: if Alice measures $a_{i}$ consistently, the distribution of $B+B^{\prime }$ (over repeated trials with *N* pairs at a time) is binomial, while the distribution of $B-B^{\prime }$ has zero variance, and vice versa in the other scenario. Thus Alice can send Bob a (superluminal) message in the classical limit.

It does not matter that the price of a one-bit message from Alice to Bob may be astronomical. As long as it is possible, at any price, it constitutes a violation of relativistic causality, which we cannot allow. Hence PR-box correlations violate relativistic causality in the classical limit, as claimed. (Note that we cannot obtain the classical limit N→∞ by setting N=∞. Rather, we take *N* finite but arbitrarily large, and for any *N*, there is no fundamental bound on the number of times Alice and Bob can repeat their measurements in order to obtain the accuracy they need for *B* and $B^{\prime }$, etc.)

Before proceeding to tripartite (GHZ) correlations, let us stop to consider bipartite quantum correlations. Does the above argument imply that they, too, allow signalling in the classical limit? If so, it cannot be correct. Most similar to PR-box correlations are quantum correlations that saturate Tsirelson’s bound [14] for the Bell-CHSH inequalities. Without loss of generality, we can consider entangled pairs of spin-1/2 particles in the state $\left[{|↑\rangle }_{A}{|↑\rangle }_{B}+{|↓\rangle }_{A}{|↓\rangle }_{B}\right]/\sqrt{2}$. In this state, Alice and Bob always obtain perfect correlations if they measure spin along the same axes in the xz plane.

Quantum correlations saturate Tsirelson’s bound when $a=\sigma_{z}^{A}$, $a^{\prime }=\sigma_{x}^{A}$, $b=\left(\sigma_{z}^{B}+\sigma_{x}^{B}\right)/\sqrt{2}$ and $b^{\prime }=\left(\sigma_{z}^{B}-\sigma_{x}^{B}\right)/\sqrt{2}$, where each of the four observables takes the values ±1. (We suppress the index *i*.) Their correlations are

(2) $$C\left(a,b\right)=C\left(a,b^{\prime }\right)=C\left(a^{\prime },b\right)=\frac{\sqrt{2}}{2}=-C\left(a^{\prime },b^{\prime }\right).$$

If Alice measures *a*, then *b* and $b^{\prime }$ are correlated with her results. If she measures $a^{\prime }$, then *b* is correlated with her results and $b^{\prime }$ is anticorrelated. Can Bob thus detect what Alice measures? As in the discussion of PR-box correlations, we can compute and compare the variances of $\left(b+b^{\prime }\right)/\sqrt{2}$ vs. $\left(b-b^{\prime }\right)/\sqrt{2}$. But, by definition, these observables correspond to $\sigma_{z}^{B}$ and $\sigma_{x}^{B}$, respectively, i.e., to *a* and $a^{\prime }$ on Bob’s particle in the pair, which is left in the same state as Alice’s. Now if Alice measures *a* consistently on her particles and Bob measures $\left(b+b^{\prime }\right)/\sqrt{2}$, the variance in Bob’s results is maximal just because the variance in Alice’s results is maximal. (That is, she has equal probability to obtain ±1). Conversely, if Alice measures *a* consistently on her particles and Bob measures $\left(b-b^{\prime }\right)/\sqrt{2}$, the variance in Bob’s results is maximal simply because a measurement of $\sigma_{x}^{B}$ after Alice measures *a* is equally likely to be ±1, whatever Alice obtains. We thus find that the correlations in Equation (2) are not strong enough to induce any difference between the variances of the observables $B+B^{\prime }$ and $B-B^{\prime }$. Indeed, they are the strongest correlations that do not induce such a difference and therefore do not permit signalling in the classical limit [10,11].

Reference [9] claims that correlations that are too strong violate relativistic causality in the classical limit, and that PR-box correlations are too strong because they provide absolute “all or nothing” correlations. But quantum mechanics, as well, provides “all or nothing” correlations. Consider a triplet of spin-half particles in a GHZ state $|\Psi_{GHZ}\rangle =\left[{|↑\rangle }_{A}{|↑\rangle }_{B}{|↑\rangle }_{J}-{|↓\rangle }_{A}{|↓\rangle }_{B}{|↓\rangle }_{J}\right]/\sqrt{2}$ shared by Alice, Bob and Jim in their respective laboratories. Suppose that these observers measure either $\sigma_{x}$ or $\sigma_{y}$ on their respective particles. Let $a_{x}$ denote Alice’s outcome from a measurement of $\sigma_{x}^{A}$ (the *x* component of the spin of her particle) and let $a_{y}$ denote Alice’s outcome from a measurement of $\sigma_{y}^{A}$ (the *y* component of the spin), with analogous notations for Bob and Jim. The state $|\Psi_{GHZ}\rangle$ is an eigenstate of the following four operators, satisfying

(3) $$\begin{array}{ccc} |\Psi_{GHZ}\rangle & = & \sigma_{y}^{A}\sigma_{x}^{B}\sigma_{y}^{J}|\Psi_{GHZ}\rangle \\ & = & \sigma_{y}^{A}\sigma_{y}^{B}\sigma_{x}^{J}|\Psi_{GHZ}\rangle \\ & = & \sigma_{x}^{A}\sigma_{y}^{B}\sigma_{y}^{J}|\Psi_{GHZ}\rangle \\ & = & -\sigma_{x}^{A}\sigma_{x}^{B}\sigma_{x}^{J}|\Psi_{GHZ}\rangle . \end{array}$$

The implication is that if all three observers measure $\sigma_{x}$ on their particles, they will discover that $a_{x}b_{x}j_{x}=-1$. Similarly, if the appropriate measurements are carried out, they will discover that $a_{x}b_{y}j_{y}=1=a_{y}b_{x}j_{y}=a_{y}b_{y}j_{x}$ as in Equation (3). In their famous paper [13], Greenberger, Horne and Zeilinger (GHZ) used these facts to show that there is no way to assign simultaneous values consistently to all six variables $a_{x}$, $a_{y}$, $b_{x}$, $b_{y}$, $j_{x}$ and $j_{y}$. This fact rules out any local hidden variable model for the GHZ state.

Can Alice, Bob and Jim use GHZ states to signal? For definiteness, let us assume that Jim tries to send a signal to Alice and Bob via his choice of what to measure, $\sigma_{x}^{J}$ or $\sigma_{y}^{J}$. Before going to the classical limit, let’s ask whether Jim can send Alice and Bob a signal using just a few triplets. Note that if Jim measures $\sigma_{x}^{J}$ and gets $j_{x}=-1$, then $a_{x}$ and $b_{x}$ must be correlated; we write $a_{x}b_{x}=1$. In the same notation, $a_{y}b_{y}=-1$. In fact, if Jim measures $\sigma_{x}^{J}$, we find $a_{x}b_{x}=-a_{y}b_{y}$ whatever he gets. On the other hand, if Jim measures $\sigma_{y}^{J}$, we obtain the analogous equation $a_{x}b_{y}=a_{y}b_{x}$, whatever he gets, and no correlation between $a_{x}$ and $b_{x}$ or $a_{y}$ and $b_{y}$. Are these correlations of any use? Alice and Bob cannot measure all their observables $a_{x},a_{y},b_{x},b_{y}$ to infer Jim’s choice.

But the commutation relations

(4) $$\left[\sigma_{x}^{A}\sigma_{x}^{B},\;\sigma_{y}^{A}\sigma_{y}^{B}\right]=0=\left[\sigma_{x}^{A}\sigma_{y}^{B},\;\sigma_{y}^{A}\sigma_{x}^{B}\right],$$

imply that Alice and Bob can obtain $a_{x}b_{x}$ and $a_{y}b_{y}$ to see if they are anticorrelated or, alternatively, can obtain $a_{x}b_{y}$ and $a_{y}b_{x}$ to see if they are correlated! In the first case, Jim must have measured $\sigma_{x}^{J}$ and in the second case, he must have measured $\sigma_{y}^{J}$. Right?

Wrong. This scheme fails. To see why, we first note that if Alice and Bob measure both $\sigma_{x}^{A}\sigma_{x}^{B}$ and $\sigma_{y}^{A}\sigma_{y}^{B}$, they will certainly find that $a_{x}b_{x}=-a_{y}b_{y}$ simply because the product of operators $\sigma_{x}^{A}\sigma_{x}^{B}\sigma_{y}^{A}\sigma_{y}^{B}$ equals $-\sigma_{z}^{A}\sigma_{z}^{B}$, which yields −1 when applied to $|\Psi_{GHZ}\rangle$. Likewise, if Alice and Bob measure both $\sigma_{x}^{A}\sigma_{y}^{B}$ and $\sigma_{y}^{A}\sigma_{x}^{B}$, they will verify that $a_{x}b_{y}=a_{y}b_{x}$, simply because the product of operators $\sigma_{x}^{A}\sigma_{y}^{B}\sigma_{y}^{A}\sigma_{x}^{B}$ equals $\sigma_{z}^{A}\sigma_{z}^{B}$, which yields 1 when applied to $|\Psi_{GHZ}\rangle$. In fact, Alice and Bob can learn nothing about Jim’s choice from their measurements.

We are back to square one. So let us try to apply the classical-limit argument of Ref. [9]. By analogy with Ref. [9], let Alice, Bob and Jim make collective measurements on ensembles of *N* triplets at a time, with Jim measuring either $\sigma_{x}^{J}$ or $\sigma_{y}^{J}$ consistently on his particles. For large enough *N*, we can define a collective variable $J_{x}=\sum j_{x}/N$, if Jim chooses to measure $\sigma_{x}^{J}$, or alternatively $J_{y}=\sum j_{y}/N$, if he chooses to measure $\sigma_{y}^{J}$, where the $j_{x}$ and $j_{y}$ represent Jim’s particles in any given ensemble. (As before, we suppress the index *i*.) We can then define also the collective variables $A_{x}=\sum a_{x}/N$, $A_{y}=\sum a_{y}/N$, $B_{x}=\sum b_{x}/N$ and $B_{y}=\sum b_{y}/N$. In some (rare) cases, one or more of these collective variables may even reach ±1. Above we noted that, for a given triplet of particles, Alice and Bob cannot measure all their observables $a_{x}$, $a_{y}$, $b_{x}$ and $b_{y}$ to infer Jim’s choice. But, according to the classical-limit argument, there cannot be such complementary between $A_{x}$ and $A_{y}$, or between $B_{x}$ and $B_{y}$. Alice and Bob must have access to at least some information about all these variables. True, their expectation values all vanish, but if Alice, Bob and Jim repeat their measurements exponentially many times, they will find fluctuations as large as ±1. Since Equation (3) involves products, we cannot directly sum over it to get a relation between $A_{x}$ or $A_{y}$ and $B_{x}$, $B_{y}$, $J_{x}$ and $J_{y}$. Even so, suppose Jim measures $\sigma_{x}^{J}$ and obtains $j_{x}=-1$ for every particle in his ensemble. Then for each of the other two particles in the triplet, $a_{x}$ and $b_{x}$ are correlated and $a_{y}$ and $b_{y}$ are anticorrelated. But Alice and Bob will not be able to detect this correlation unless another “miracle” occurs, in addition to the “miracle” that happened in Jim’s laboratory. For example, suppose that $A_{x}=1$. It follows from Equation (3) that $B_{x}=1$ (up to fluctuations due to measurement errors). Then Alice and Bob could compare their results for $A_{x}$ and $B_{x}$ to uncover a striking correlation between them and conclude that Jim had measured $J_{x}$ and not $J_{y}$.

But this conclusion can be valid only if the statistics support it. In this scenario, we have assumed rare fluctuations: $J_{x}=-1$ and $A_{x}=1$. Since the two fluctuations are independent, their combined probability is the product of their individual probabilities, namely $2^{-N}\times 2^{-N}=2^{-2N}$. For this rare scenario, we don’t need to assume also that $B_{x}=1$; Equation (3) requires it. Thus, with probability $2^{-2N}$, Alice and Bob will obtain $A_{x}=1=B_{x}$. Does this result imply that Jim consistently measured $\sigma_{x}^{J}$ on his particles? How likely is it that Alice and Bob would have obtained $A_{x}=1$ and $B_{x}=1$ if Jim had chosen to measure $\sigma_{y}^{J}$ on all his particles, making $a_{x}$ and $b_{x}$ uncorrelated? The probability would have been $2^{-2N}$, exactly the same. So, once again, Alice and Bob have no way of reading Jim’s one-bit message (his choice of what to measure). Likewise, Alice and Bob can try to signal to Jim by, say, measuring $\sigma_{x}^{A}=\sigma_{x}^{B}$. If they get $A_{x}=1=B_{x}$, Jim will certainly obtain $J_{x}=-1$. But the probability that Jim will obtain $J_{x}=-1$ by chance is $2^{-N}$, at least as large as the probability $2^{-2N}$ that Alice and Bob will obtain $A_{x}=1=B_{x}$ or even the probability $2^{-N}$ that Alice and Bob will obtain $\sigma_{x}^{A}\sigma_{x}^{B}=1$ for all the *N* pairs in their ensemble.

The statistics don’t work out in the case of GHZ triplets as they do in the case of PR-box pairs. We therefore conclude that despite the similarity between Equations (1) and (3), GHZ correlations do not allow Jim to signal to Alice and Bob by choosing which observable to measure (at least via the above attempts), even if we assume a classical limit in which they can measure the ensemble averages of incompatible observables. The argument of Ref. [9] passes the test we prepared for it.

## 2. Retrocausality in PR-Box and GHZ Correlations

**Figure 1:**
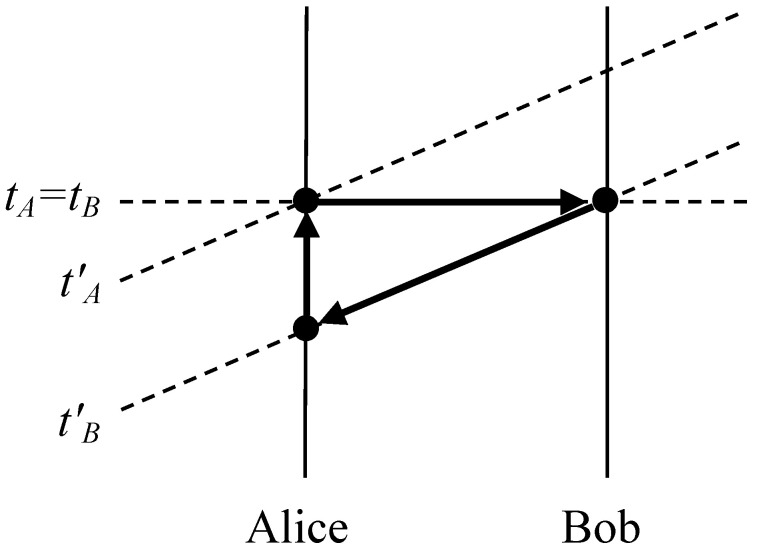
The horizontal dotted line represents an equal-time surface in the unprimed frame, while the tilted dotted lines represent two equal-time surfaces in the primed frame. The arrows, each representing a cause and an effect, form a closed causal loop.

Instantaneous signalling directly violates relativity theory, opening the door to causal loops and contradictions. In particular, consider the classical limit of a PR-box ensemble, with Alice sending one bit of information $i_{A}\in \left\{0,1\right\}$ to distant Bob. In an “unprimed” reference frame, Bob receives Alice’s message instantaneously (at time $t_{B}=t_{A}$); but in an appropriate “primed” reference frame, Alice’s bit could be a message into the past, e.g., Bob receives her bit (at time $t_{B}^{\prime }$) before she sends it (at time $t_{A}^{\prime }>t_{B}^{\prime }$). Applying the principle of relativity, we infer that in the primed reference frame, Bob could send a bit $i_{B}\in \left\{0,1\right\}$ at time $t_{B}^{\prime }$ that Alice would receive instantaneously (at time $t_{B}^{\prime }$) before sending $i_{A}$. Then if Alice’s device is set to echo whatever message she receives from Bob (so that $i_{A}=i_{B}$), and Bob’s device is set to yield the inverse of the message he receives from Alice (so that $i_{B}=1-i_{A}$), together they create a self-contradictory causal loop, as in Figure 1.

From this example it may seem obvious that PR-box correlations and GHZ correlations are distinguished, in that PR-box correlations in the classical limit can be retrocausal, and create self-contradictory causal loops, whereas GHZ correlations cannot be retrocausal. It is therefore of interest to note that this distinction is not valid. GHZ correlations can be understood as retrocausal, as well! Yet the predictions implied by Equation (3) do not create causal loops. How can quantum correlations affect distant or past events without creating causal loops?

**Figure 2:**
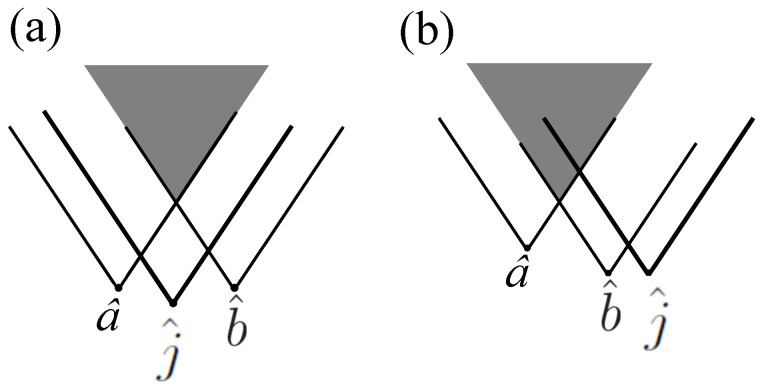
The overlap of the future light cones of $\hat{a}$ and $\hat{b}$ either (**a**) lies or (**b**) does not lie entirely within the future light cone of $\hat{j}$.

Reference [15] imagines an action called “jamming” in which Jim “the Jammer” can, by pushing a button on a device he holds, decide at any moment whether to turn an ensemble of entangled pairs of particles shared by Alice and Bob into a product state. Although jamming is action at a distance, it is consistent with relativistic causality if two conditions are met. The first condition, the unary condition, states that Alice and Bob cannot infer Jim’s decision from the results of their separate measurements. For example, if—regardless of Jim’s decision—Alice measures either *a* or $a^{\prime }$, and obtains results ±1 with equal probability, and likewise Bob measures either *b* or $b^{\prime }$, and obtains results ±1 with equal probability, then the unary condition is fulfilled. The binary condition states that if $\hat{a}$ is the spacetime event of Alice’s measurements on her ensemble, $\hat{b}$ is the spacetime event of Bob’s measurements on his ensemble, and $\hat{j}$ is the spacetime of event of Jim pushing the button on his device, then the overlap of the forward light cones of $\hat{a}$ and $\hat{b}$ lies entirely within the forward light cone of $\hat{j}$. (See Figure 2). As shown in Ref. [15], if jamming obeys the unary and binary conditions, then it is consistent with relativistic causality even though $\hat{a}$ and $\hat{b}$ may be *earlier* in time than $\hat{j}$. While jamming is natural in the context of quantum information theory, in Ref. [15] it provides an example of how a nonlocal equation of motion can be consistent with the no-signalling constraint.

We return now to the GHZ correlations of Equation (3) and show that they permit jamming [16]. Suppose Alice, Bob and Jim share an ensemble of particle triplets in the GHZ state. If Jim consistently measures $\sigma_{z}^{J}$, he disentangles Alice’s particles from Bob’s, regardless of the outcomes he gets. If he measures $\sigma_{x}^{J}$, Alice’s particles remain entangled with Bob’s particles, and their spins are correlated. For example, $\sigma_{x}^{A}$ and $\sigma_{x}^{B}$ are perfectly correlated or perfectly anticorrelated, depending on Jim’s outcome. If the information regarding Jim’s outcomes is delivered to Alice and Bob, they can bin their $\sigma_{x}$ measurements in two ensembles corresponding to Jim’s outcomes ±1. They will find that their results, within each ensemble, are perfectly (anti-)correlated in the case that Jim had chosen to measure $\sigma_{x}^{J}$, or uncorrelated in case he had measured $\sigma_{z}^{J}$.

**Figure 3:**
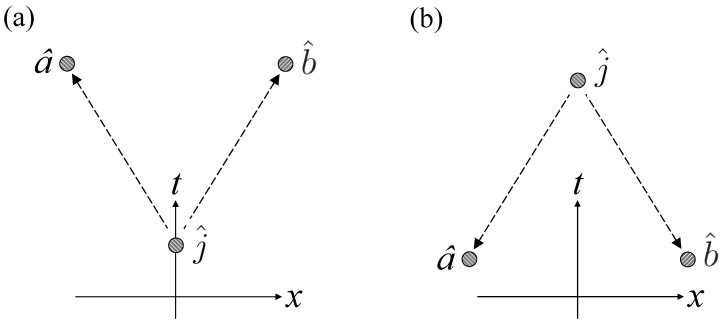
Configurations in which Jim can (**a**) causally and (**b**) retrocausally put pairs of particles shared by Alice and Bob in product or entangled states, as he chooses. The dashed arrows connect cause with effect.

This realization of jamming satisfies the unary condition because, regardless of Jim’s decision, Alice’s measurements of $\sigma_{x}^{A}$ average to zero, and likewise for Bob’s measurements of $\sigma_{x}^{B}$. It fulfills the binary condition because Jim must report to Alice and Bob the results of his measurements of $\sigma_{z}^{J}$ or $\sigma_{x}^{J}$ for them to determine, from the results of *their* measurements, whether their pairs were entangled or not. Now, Alice and Bob can make their determination *only* in the overlap of the future light cones of $\hat{a}$ and $\hat{b}$, which must lie in the future light cone of $\hat{j}$ for them to receive Jim’s input. Thus jamming via GHZ triplets is consistent with relativistic causality. Nevertheless, Jim’s decision, whether to leave the pairs shared by Alice and Bob in entangled or product states, can take place even *later* than $\hat{a}$ and $\hat{b}$, and even at a timelike separation from both measurements $\hat{a}$ and $\hat{b}$. (See Figure 3). Even then, it is only in the forward light cone of $\hat{j}$ that Alice and Bob can combine their data and determine whether Jim jammed their measurements.

So what makes PR-box correlations different from GHZ correlations, such that the former violate relativistic causality (in the classical limit) while the latter do not? We might have replied, “PR-box correlations are retrocausal whereas GHZ correlations are not”. But we have just seen that this distinction fails. So let us return to our comparison, in the first section, of PR-box correlations and bipartite quantum correlations. We noted that even quantum correlations that violate the Bell-CHSH inequality maximally are not strong enough to permit signalling. Are GHZ correlations, which like PR-box correlations can be 0 or 1, strong enough? No! They are indeed stronger, but their strength dissipates over the *two* stages Alice and Bob require in attempting to receive Jim’s signal. Relativistic causality in the classical limit is a subtle, but effective, constraint on quantum mechanics.

We introduced this work by stating that three axioms with clear physical meaning, namely nonlocality, relativistic causality, and the existence of a classical limit, might be sufficient for deriving quantum mechanics, or at least an important part of the theory. We can consider reducing these three axioms to two simply by eliminating nonlocality as an axiom. Indeed, axioms in physical theories are, in general, constraints. The constraint of locality could be an axiom, but absence of this constraint need not be an axiom. And it seems from our work that quantum mechanics is just as nonlocal as it can be without violating relativistic causality. The retrocausality we have seen in jamming via GHZ correlations suggests that also retrocausality, like nonlocality, can appear wherever it is not forbidden by relativistic causality.
